# National Institutes of Health Stroke Scale (NIHSS) on admission predicts acute symptomatic seizure risk in ischemic stroke: a population-based study involving 135,117 cases

**DOI:** 10.1038/s41598-020-60628-9

**Published:** 2020-03-02

**Authors:** Johann Philipp Zöllner, Björn Misselwitz, Manfred Kaps, Marco Stein, Jürgen Konczalla, Christian Roth, Karsten Krakow, Helmuth Steinmetz, Felix Rosenow, Adam Strzelczyk

**Affiliations:** 1 Department of Neurology and Epilepsy Center Frankfurt Rhine-Main, Goethe-University Frankfurt, Frankfurt am Main, Germany; 2 LOEWE Center for Personalized Translational Epilepsy Research (CePTER), Goethe-University Frankfurt, Frankfurt am Main, Germany; 3 Institute of Quality Assurance Hessen (Geschäftsstelle Qualitätssicherung Hessen [GQH]), Eschborn, Germany; 4 Department of Neurology, Justus-Liebig-University Giessen, Giessen, Germany; 5 Department of Neurosurgery, Justus-Liebig-University Giessen, Giessen, Germany; 6 Department of Neurosurgery, Goethe-University Frankfurt, Frankfurt am Main, Germany; 7 Department of Neurology, DRK-Kliniken Nordhessen, Kassel, Germany; 8 Department of Neurology and Epilepsy Center Hessen, Philipps-University Marburg, Marburg (Lahn), Germany; 9 Asklepios Neurologische Klinik Falkenstein, Königstein-Falkenstein, Germany

**Keywords:** Neurology, Risk factors

## Abstract

The National Institutes of Health Stroke Scale (NIHSS) score is the most frequently used score worldwide for assessing the clinical severity of a stroke. Prior research suggested an association between acute symptomatic seizures after stroke and poorer outcome. We determined the frequency of acute seizures after ischemic stroke in a large population-based registry in a central European region between 2004 and 2016 and identified risk factors for acute seizures in univariate and multivariate analyses. Additionally, we determined the influence of seizures on morbidity and mortality in a matched case–control design. Our analysis of 135,117 cases demonstrated a seizure frequency of 1.3%. Seizure risk was 0.6% with an NIHSS score at admission <3 points and increased up to 7.0% with >31 score points. Seizure risk was significantly higher in the presence of acute non-neurological infections (odds ratio: 3.4; 95% confidence interval: 2.8–4.1). A lower premorbid functional level also significantly increased seizure risk (OR: 1.7; 95%CI: 1.4–2.0). Mortality in patients with acute symptomatic seizures was almost doubled when compared to controls matched for age, gender, and stroke severity. Acute symptomatic seizures increase morbidity and mortality in ischemic stroke. Their odds increase with a higher NIHSS score at admission.

## Introduction

Ischemic stroke has long been recognized as one of the major risk factors for the development of structural epilepsy, particularly among the elderly^1^. In the elderly population, strokes may represent a cause of epilepsy in more than 30% of cases^2,3^.

Epileptic seizures can occur early (known as acute symptomatic seizures) or late after a stroke, with the threshold usually set at seven days^4^. Between 1.1% and 10% of stroke patients end up suffering from acute symptomatic seizures, while 3% to 25.2% develop poststroke epilepsy^1,5–12^. While the occurrence of acute symptomatic seizures during the stroke episode can predict the occurrence of poststroke epilepsy^13^, late seizures in patients with a structural lesion due to the stroke by definition result in the diagnosis of epilepsy^6,14^.

The risk factors for the occurrence of acute symptomatic seizures after ischemic stroke have been investigated in several studies to date and include anterior hemisphere location, cortical localization, stroke severity, and hemorrhagic transformation^6,7,10,15,16^. Previously, stroke severity was assessed by using the Scandinavian Stroke Scale (SSS), Canadian Neurological Score (CNS), modified Rankin Scale (mRS) at admission and the NIHSS in two smaller studies^6,7,15–18^. Predictive scores have also been constructed for the occurrence of late seizures following stroke and poststroke epilepsy^19,20^. The majority of relevant studies have prospectively analyzed a comparatively small number of patients, so there exists a dearth of large population-based studies. Further, the influence of acute symptomatic seizures on mortality is controversial^6,8,21,22^.

The aim of this study was to investigate the frequency of acute symptomatic seizures following ischemic stroke in a defined, large population in central Europe and to determine if and to what extent a higher National Institutes of Health Stroke Scale (NIHSS) score at admission predicts seizure risk. In a second step, we assessed risk factors for the occurrence of acute symptomatic seizures. Furthermore, we examined the influence of acute symptomatic seizures on mortality in a matched case–control analysis. Patients with acute symptomatic seizures in intracerebral and subarachnoid hemorrhage were evaluated in an independent study^23^. 

## Results

The GQH database (Institute of Quality Assurance Hessen, Eschborn, Germany) contains information on 297,120 patients with ischemic stroke, transient ischemic attack, and intracerebral and subarachnoid hemorrhage collected between January 2004 and December 2016. After application of the inclusion criteria for ischemic stroke, 135,117 patients remained. During the evaluation period, acute symptomatic seizures occurred in a total of 1,787 (1.3%) ischemic stroke patients. Seizure incidence did not change significantly between 2004 and 2016 [average annual percentage change: −1.5%, 95% confidence interval (CI): −5.5 to 2.7; *p* = 0.5]. In total, 66,157 patients had an NIHSS score at admission of zero to five and 40,005 had an NIHSS score of more than five. For detailed results, please refer to Table 1.

**Table 1:** Frequency of acute symptomatic seizures and clinical differences between patients with and without seizures.

Ischemic stroke patients				
	Seizures (n = 1.787)	No Seizures (n = 133.330)	P*	*% valid records*
*Mean age, years*	75.4 (SD 12.4)	73.9 (SD 12.7)	**0.001**	100
*Female gender, %*	53.7	49.7	**0.001**	100
*Arterial hypertension, %*	82.2	79.9	0.110	89.2
*Diabetes mellitus, %*	27.9	27.3	0.541	60.1
*Hypercholesterolemia, %*	21.2	24.5	**0.037**	42.0
*Atrial fibrillation, %*	36.3	24.8	**<0.001**	58.9
*Smoker within the last 10 years*	7.5	8.9	0.305	36.5
*Preexisting stroke, %*	32.5	22.4	**<0.001**	58.1
*Acute infection, %*	37.5	11.9	**<0.001**	72.7
*Preexisting anticoagulation, %*	13.3	10.2	**<0.001**	100
*Preexisting antiplatelets, %*	45.6	42.8	**0.023**	100
*mRS score before event of 3–5, %*	28.1	14.8	**<0.001**	91.0
*mRS score <24 h after admission of 3–5, %*	57.5	38.3	**<0.001**	99.5
*mRS score at discharge of 3–5, %*	85.1	58.0	**<0.001**	93.9
*Mean NIHSS score at admission*	11.7 (SD 8.5)	6.0 (SD 6.3)	**<0.001**	79.0
*Mean NIHSS score at discharge*	6.9 (SD 9.0)	2.9 (SD 5.2)	**<0.001**	6.9

ICU = intensive care unit, mRS = modified Rankin Scale, NIHSS = National Institutes of Health Stroke Scale, *P-values adjusted using the Benjamini-Hochberg false discovery rate method.

### Univariate and multivariate analysis of risk factors

Patients with ischemic stroke who suffered an acute symptomatic seizure were typically significantly older (75.4 vs. 73.9 years; *p* = 0.001) and more often female (53.7%; *p* = 0.001). Further, they were significantly more likely to have atrial fibrillation and to have had a prior stroke. Hypercholesterolemia was less common in ischemic stroke patients with seizures. Seizure-free patients significantly more often received statins as secondary prophylaxis (55.7% vs. 41.7%; *p* < 0.001). For detailed results of the univariate analysis, please refer to Table 1. Acute non-neurological infections were significantly more common in patients with acute symptomatic seizures (37.5% vs. 11.9%; *p* < 0.001). These individuals were more dependent in everyday life (mRS score > 2) even before admission and suffered more severe strokes (mean NIHSS score: 11.7 vs. 6.0; *p* < 0.001). More than three-quarters (85.1%) of patients with acute symptomatic seizures were dependent on external aid in their daily lives (mRS score > 2) once they left the hospital. In comparison, this was true in less than two-thirds of patients who did not experience an acute symptomatic seizure (58.0%; *p* < 0.001). Patients with ischemic stroke and acute symptomatic seizures were also more often pretreated with anticoagulants and antiplatelets (*p* < 0.001 and *p* = 0.023).

**Figure 1:**
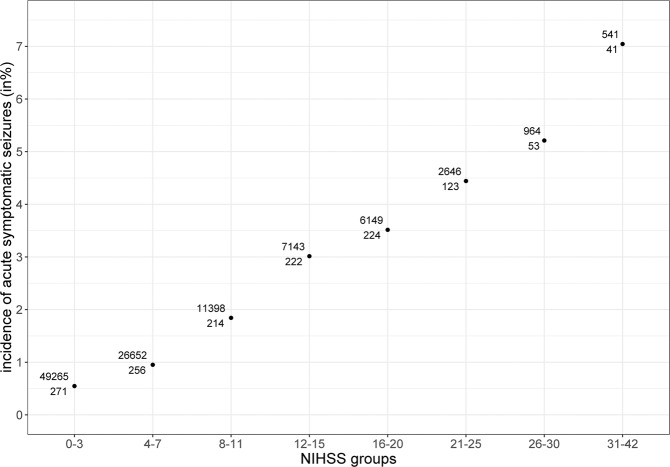
The bottom number shows the count of patients with acute symptomatic seizures in this NIHSS range (x-axis), while the upper number shows the count of patients without seizures. NIHSS = National Institutes of Health Stroke Scale.

In univariate regression analysis, seizure risk was 0.6% in those with an NIHSS score of zero points, and the odds increased by 9.2% for every one-point increase in the NIHSS (exp(b): 1.092; 95% CI: 1.086–1.098; *p* < 0.001). In those with an NIHSS score of more than 31 points, the seizure risk was 7.0% (Fig. 1).

**Figure 2:**
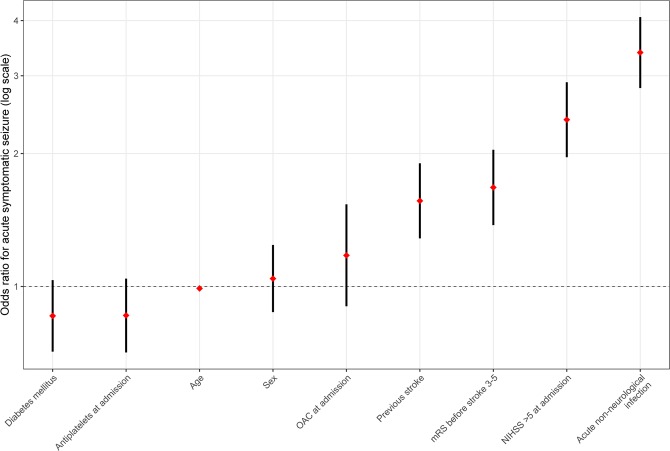
Risk factors are sorted by ascending ORs. Red diamonds indicate ORs; vertical black bars indicate 95% confidence intervals. mRS = modified Rankin Scale, NIHSS = National Institutes of Health Stroke Scale, OAC = oral anticoagulation.

The multivariate regression analysis (n = 39,573 patients with complete datasets) confirmed acute non-neurological infection to be an independent risk factor for the occurrence of acute symptomatic seizures, with an odds ratio (OR) of 3.4 (95% CI: 2.8–4.1; *p* < 0.001). An NIHSS score of more than five points was also associated with seizures (OR: 2.4, 95% CI: 2.0–2.9; *p* < 0.001), as was having a premorbid mRS score of three to five points (OR: 1.7, 95% CI: 1.4–2.0; *p* < 0.001) or having suffered a previous stroke (OR: 1.6, 95% CI: 1.3–1.9; *p* < 0.001). Advancing age resulted in a lower risk (−1% odds change/year, OR: 0.99, 95% CI: 0.98–1.0; *p* = 0.008). Patients’ sex (OR: 1.0; 95% CI: 0.9–1.2; *p* = 0.643) and a history of diabetes mellitus (OR: 0.9; 95% CI: 0.7–1.0; *p*=0.107) did not promote a significant change. Pre-existing treatment with anticoagulants (OR: 1.2; 95% CI: 0.9–1.5; *p* = 0.232) or antiplatelets (OR: 0.9; 95% CI: 0.7–1.0; *p* = 0.124) also had no significant effect on the change in odds. A secondary analysis with NIHSS as a numeric predictor from the same dataset (n = 39,573) did not change significances. For the full model, see Table 2. For visual representation, please refer to Fig. 2.

**Table 2:** Results of the logistic multivariate regression analysis to determine occurrence of acute symptomatic seizures in ischemic stroke patients.

	*Regression Coefficient (Standard Error)*	95% CI - lower bound	Adjusted odds ratio (exp *b*)	95% CI - upper bound
*Constant*	−4.493		0.011	
Age (years)	−0.010* (0.004)	0.983	0.990	0.997
Sex (female)	0.041 (0.089)	0.875	1.042	1.242
Diabetes mellitus	−0.153 (0.095)	0.712	0.858	1.034
Previous stroke	0.446** (0.100)	1.285	1.563	1.901
Pre-treatment anticoagulants	0.13 (0.136)	0.902	1.176	1.535
Pre-treatment antiplatelets	−0.151 (0.098)	0.709	0.860	1.042
Pre-morbid mRS 3-5	0.516** (0.100)	1.377	1.676	2.040
NIHSS >5	0.870** (0.100)	1.962	2.386	2.901
Acute infection	1.220** (0.094)	2.814	3.386	4.075
Nagelkerke’s *R*^2^ = 0.081				

* indicates statistical significance at P < 0.05, ** indicates significance at P < 0.001, 95% CI = 95% confidence interval for the adjusted odds ratio (exp *b*). mRS = modified Rankin Scale, NIHSS = National Institutes of Health Stroke Scale.

### Case–control analysis

**Figure 3:**
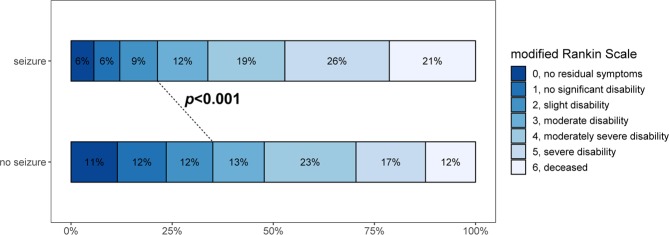
The modified Rankin Scale (mRS) score at discharge demonstrates a shift toward worse outcomes for patients with acute symptomatic seizures as compared with those without.

In patients with ischemic stroke who were matched for age, sex, premorbid functional level, and stroke severity, the occurrence of acute seizures was associated with a significantly increased rate of intensive care unit (ICU) admission (16.1% vs. 12.4%; *p* < 0.001). Of note, the necessity for mechanical ventilation was also higher among patients with acute seizures (13.2% vs. 8.4%; *p* = 0.045). In-hospital mortality was almost twice as high in patients with acute symptomatic seizures (27.0% vs. 14.1%; *p* < 0.001). Morbidity as expressed on the mRS at discharge was significantly higher in seizure patients, as demonstrated in Fig. 3 [median mRS score: 4.0 in both (SD 1.8 and SD 1.9); p < 0.001].

## Discussion

Acute symptomatic seizures are an important complication after stroke and are associated with an increased risk of post-stroke epilepsy. Immediate sequelae of acute symptomatic seizures, including aspiration pneumonia and status epilepticus^24,25^, can be severe. Our study aimed to analyze the frequency of acute symptomatic seizures following ischemic stroke in a large, defined population in central Europe and to evaluate seizure predictors as well as the influence on morbidity and mortality.

Our most important findings are that the risk of acute symptomatic seizures increases with increasing NIHSS score at admission, with the odds increasing by 9.2% for every one-point increase. This may explain the divergent incidence results in previous studies^7,17,22^. Additionally, we found that seizure risk is increased in the context of acute non-neurological infection (e.g., pneumonia, urinary tract infection, sepsis). We confirm earlier findings that a lower premorbid functional level, where the patient is already at least partially dependent on external help, also increases the risk of acute symptomatic seizures^8^. We further demonstrate that acute symptomatic seizures are associated with increased morbidity and mortality rates in ischemic stroke at hospital discharge, even after adjusting for stroke severity, pre-morbid functional status, age, and gender. Ischemic stroke patients with acute symptomatic seizures were also more likely to be admitted to the ICU and require mechanical ventilation than controls matched for stroke severity, pre-morbid functional status, age, and gender.

The ENCHANTED study identified febrile temperatures as a risk factor for acute seizures^26^, while a Norwegian register study found a relationship between NIHSS score, acute infection, and acute seizures^17^. Our study confirms several previously reported risk factors for acute symptomatic seizures such as more severe stroke and lower functional status at admission^6,16,17^. As in other studies^18,21^, we found a smaller risk of acute seizures in older ischemic stroke patients, although this effect was small in our study (−1% odds change/year). The causes for this striking finding cannot be inferred from our results, they might relate to changes in excitability or less frequent cerebral oedema in older patients. Interestingly, while pre-treatment with oral anticoagulants and antiplatelet agents was significantly more common in ischemic stroke patients with seizures in the univariate analysis, this did not increase the odds of suffering an acute symptomatic seizure in the multivariate analysis. Hypercholesterolemia was more frequent in the non-seizure group and the cause cannot be inferred from our database. Interestingly, while hypercholesterolemia was also less frequent in patients with haemorrhagic stroke in another study^27^, further studies in ischemic stroke failed to find significant differences between patients with and without seizures^6^.

Previous studies have suggested divergent results regarding the influence of acute seizures on mortality in ischemic stroke. One study demonstrated an increased long-term mortality in stroke patients with seizures but only included patients suffering from status epilepticus^25^. One of the first studies on seizure-related deaths in stroke patients found an increased mortality but was not adjusted for stroke severity^6^. A subsequent study showed increased mortality in patients with seizures even after adjustment for stroke severity but did not use the NIHSS^21^. Adjustment for the NIHSS led to acute seizures not being independently associated with increased mortality in a third study^22^. Our study demonstrates in a large population-based registry that acute seizures are associated with increased mortality after adjusting for ischemic stroke severity using the NIHSS. In addition, acute symptomatic seizures prolong the length of stay in the ICU and increase the necessity for mechanical ventilation. Furthermore, they are associated with higher mRS scores at discharge, independent of premorbid functional status or NIHSS score at admission.

The mandatory inclusion of all patients in the GQH database allowed us to define a comprehensive incidence rate of acute symptomatic seizures after ischemic stroke of 1.3%, which did not change significantly within 13 years. The advantage of our large and population-based cohort of more than 135,000 patients is offset by the lack of granularity of clinical data. This implies several limitations: Apart from the retrospective study design, no data is included in the registry on several factors that have been shown to predict acute symptomatic seizures, such as stroke etiology, stroke size and (cortical) location. In addition, information on prior seizures, seizure type and existing anti-seizure medication is lacking. Electroencephalographic (EEG) information is missing as well and it is known that post-stroke seizures are underestimated without systematic implementation of EEG^28^. Some clinical information is lacking in the database, reducing the number of patients in a complete-case analysis. While we could match patients in the mortality analysis based in several important predictors of in-hospital death, some morbidity scores and information on the withdrawal of life-sustaining treatment were not included in the database and might explain partially differences in patient mortality between groups. Further cohort studies with detailed descriptions of individual patients will have to answer some of these questions. Still, our large cohort size allowed us to determine the included risk factors with greater certainty, and this constitutes the major strength of this study.

## Methods

We evaluated data from the stroke registry of the Institute of Quality Assurance Hessen [Geschäftsstelle Qualitätssicherung Hessen (GQH), Eschborn, Germany; www.gqhnet.de] over a 13-year period (2004–2016). The GQH stroke registry is used to investigate the treatment quality of patients with strokes in more than 100 clinics in Hessen, a federal state of the Federal Republic of Germany with more than six million inhabitants (www.destatis.de; 6,176,172 inhabitants on December 31, 2015). The data is entered by the treating physicians or a coding specialist and the validity of the entries is checked by regular audits. Data entry is mandatory for all hospitals in Hessen, and cross-validation with billing data according to §21 Krankenhausentgeltgesetz (the “Hospital Remuneration Act”) shows more than 99% completeness. Data analysis for research purposes was approved by the ethical committee of the medical faculty of the Goethe-University Frankfurt.

Inclusion criteria were age of 18 years or older, ischemic stroke (International Statistical Classification of Diseases and Related Health Problems, 10^th^ revision code I63.x), defined as an acute loss of neurological function due to a cerebrovascular cause persisting for more than 24 hours with exclusion of an intracerebral bleeding^23^ on an adequate imaging modality (cranial computed tomography [cCT] or cranial magnetic resonance imaging [cMRI]) or imaging findings of ischemic stroke, and admission to the hospital within 24 hours after symptom onset. We extracted age; sex; time from onset of symptoms to admission; overall incidence of epileptic seizures, presence of arterial hypertension, diabetes mellitus, hypercholesterolemia, and atrial fibrillation; the modified Rankin Scale (mRS) score; NIHSS score; the occurrence of urinary tract infection, pneumonia, or sepsis; and preexisting treatment with anticoagulants or antiplatelet agents.

In the registry, seizures are recorded prospectively during inpatient treatment, but no information is provided on seizure semiology, frequency, or exact time of occurrence. The database also does not contain any information on cortical stroke localization or stroke volume. There is no follow-up conducted beyond the hospital admission.

### Statistical methods

The mRS score was dichotomized into zero to two points (living independently) and three to five points (at least some dependency in everyday life), respectively, while the NIHSS score was dichotomized into less than or equal to five points (mild to no impairment) and more than five points (moderate to severe impairment); for a secondary analysis, the NIHSS was treated as a numerical variable. The infection variables were combined under the umbrella of “acute non-neurological infection.” We used the chi-squared test for categorical variables and the Mann–Whitney U test for continuous variables in a univariate analysis. Univariate logistic regression was employed to evaluate the relation between NIHSS score and acute symptomatic seizures. We used multivariate forced entry logistic regression to determine the occurrence of acute symptomatic seizures. Additionally, we performed a matched case–control analysis. Complete-case analyses were used exclusively. Patients were matched based on age, gender, and NIHSS score at admission as well as pre-morbid mRS score. Out of 1,787 patients, a total of 34 patients could not be matched. Statistical analyses were performed using the SPSS version 26.0 software program (IBM Corp., Armonk, NY, USA). Trend analysis was performed using JoinPoint version 4.7.0.0 (Statistical Methodology and Applications Branch, Surveillance Research Program, National Cancer Institute, Bethesda, MD, USA). Statistical significance was assumed at a *p*-value of less than 0.05. *P*-values were adjusted for multiple comparisons using the Benjamini-Hochberg false discovery rate method as implemented in the “p.adjust” function in R version 3.5.3 (R Core Team, Vienna, Austria).

## Data Availability

Data are available upon request from the Geschäftsstelle Qualitätssicherung Hessen (GQH), pending certain prerequisites.
